# AI-Enabled Personalization of Semaglutide Therapy in Type 2 Diabetes: Systematic Review With an Integration Framework

**DOI:** 10.2196/86960

**Published:** 2026-03-09

**Authors:** Ghinwa Barakat, Samer El Hajj Hassan, Hanane Akhdar, Nghia Duong-Trung, Wiam Ramadan

**Affiliations:** 1 Biological and Chemical Sciences Department School of Arts And Sciences Lebanese International University Beirut Lebanon; 2 IU International University of Applied Sciences Berlin Germany; 3 School of Engineering (ESIB) Saint Joseph University Beirut Lebanon; 4 Computer Science and Information Technology Department Lebanese International University Beirut Lebanon; 5 Faculty of Sciences V Lebanese University Nabatieh Lebanon; 6 Nutrition and Food Science Department School of Arts And Sciences Lebanese International University Beirut Lebanon

**Keywords:** artificial intelligence, clinical decision support, Ozempic, semaglutide, personalized medicine, type 2 diabetes mellitus

## Abstract

**Background:**

Type 2 diabetes mellitus (T2D) is a rapidly growing global health concern requiring innovative treatment methods. Ozempic (semaglutide), a glucagon-like peptide-1 receptor agonist, has proven consistent effectiveness in lowering blood glucose levels, supporting weight loss, and minimizing cardiovascular complications. In parallel, artificial intelligence (AI) elevates diabetes care yet complements these efforts by converting raw data from wearable devices, electronic health records, and medical imaging into practical insights for efficient, tailored, and customized treatment plans.

**Objective:**

The objective of this systematic review is to examine current evidence of AI-driven methods to optimize Ozempic-based T2D therapy.

**Methods:**

A total of 18 peer-reviewed articles were identified, revealing four dominant thematic clusters: (1) patient stratification and risk prediction, (2) AI-enhanced imaging for body composition changes, (3) cardiovascular and metabolic risk assessment, and (4) personalized AI-driven dosage.

**Results:**

Across multiple metrics, such as glycated hemoglobin reduction, weight loss, cardiovascular benefits, and adverse event mitigation, AI-based approaches outperformed standard fixed-dose regimens. A theoretical framework is proposed for AI-Ozempic integration, with continuous data collection, AI processing, clinical decision support, real-time support, and real-time feedback and modeling iteration refinement cycles.

**Conclusions:**

Significant gaps remain a persistent challenge, including the need for large-scale randomized controlled trials, longer follow-up periods, explainable AI models, regulatory validation, and practical strategies for routine clinical implementation. The findings emphasize the AI’s potential to transform semaglutide therapy while delineating important paths for future research.

## Introduction

Type 2 diabetes mellitus (T2D) has become one of the most pressing global health challenges affecting more than 400 million individuals worldwide, which impacts the health care system and economies globally [1-3]. The disease is characterized by β cells dysfunction [4]. If it is poorly managed, T2D can lead to severe microvascular and macrovascular complications, affecting the quality of life and increasing mortality risk [5,6]. Traditional medical agents for T2D, which include metformin, sulfonylurea, and insulin, have been used historically to maintain glycemic control [7]. However, these treatments are often ineffective in preventing long-term complications and may be associated with adverse effects such as hypoglycemia, weight gain, and diminished patient adherence [8,9]. In response, modern therapies, particularly glucagon-like peptide-1 (GLP-1) receptor agonists [10,11], are now widely used in diabetes management. Semaglutide, known as Ozempic, has shown significant efficacy in reducing hemoglobin A_1c_ (HbA_1c_), promoting weight loss, and providing heart protection in patients with T2D [12-14].

Traditional semaglutide therapies (injectable weekly Ozempic; daily oral Rybelsus) differ from older glucose-lowering medications (sulfonylureas or insulin) by having high HbA_1c_-lowering efficacy, clinically significant weight loss, and lower intrinsic hypoglycemia risk when used without insulin or sulfonylureas [15]. GLP-1 receptor agonists, such as semaglutide, are now commonly recommended first, according to current guidelines, when weight loss and/or cardiovascular risk reduction are considered important factors. This is due to the potential for these drugs to provide more “multibenefit” options compared to some of the legacy agents, which were primarily focused on glycemia only [15]. As far as the actual prescribing practices are concerned, there has been a marked increase in the number of prescriptions written for GLP-1 receptor agonists over the last few years. Additionally, semaglutide products are being prescribed in a high percentage of patients in obesity-adjacent populations, which is reflective of the drug’s current high usage and popularity [16]. Regarding the risks associated with semaglutide, the primary concerns have centered on dose-dependent gastrointestinal side effects, and rare but clinically relevant issues, such as pancreatitis, have also become a concern. Thus, the advance in efficacy with semaglutide has been largely achieved through tolerability and monitoring trade-offs and not through an increase in the severity of side effects [15]. Ozempic (semaglutide) is taken as part of a well-planned, multistep ”dose-escalation” program to help the body get used to it. It starts with an initial nonmedication use dose of 0.25 mg per week for 4 weeks and then goes up to 0.5 mg per week after that, with the potential to take 1.0 mg per week based on how well the patient tolerates the drug [17]. This gradual titration strategy is designed to optimize efficacy while minimizing dose-dependent gastrointestinal adverse effects and represents a standard clinical approach for GLP-1 receptor agonist therapy.

In parallel, the adoption of artificial intelligence (AI) technologies, including machine learning (ML), deep learning (DL), and predictive analytics, is reshaping the health care delivery and clinical decision-making processes [18]. AI tools use extensive data sets consisting of electronic health records (EHRs), medical imaging, wearable devices, and omics-based data to tailor medical interventions to individual patient profiles. Recent evidence indicates that AI-driven models can accurately predict patient response to GLP-1 receptor agonist therapy [19,20], identify clinically significant changes in body composition through advanced imaging analysis, and improve cardiovascular risk assessment [21,22]. Such predictive resources allow for more accurate therapeutic decisions, improving short-term results and long-term disease management [23]. Although the broader role of AI in diabetes management has attracted considerable interest in research, studies specifically focused on the integration of AI with Ozempic treatment remain comparatively scarce. Several investigations have used AI techniques for general diabetes care or have been widely applied to several glucose-lowering therapies [24,25]. However, very few studies explicitly examine the potential opportunities and challenges of combining AI with Ozempic to customize dosage schedules, predict therapeutic response, and monitor real-world efficacy [26,27]. Previous reviews have explored AI applications in diabetes care broadly [24,25] or focused on GLP-1 receptor agonists without an AI lens [28]. None has synthesized AI techniques specifically for semaglutide, nor evaluated methodological quality with a tailored Customized Quality Assessment Scale (CQAS) instrument. This clear research gap highlights the need for a systematic review dedicated to synthesizing existing evidence, identifying prevailing topics, and proposing an evidence-based conceptual structure that guides the future integration of AI technologies into personalized Ozempic treatment for T2D. Thus, the following three objectives are addressed in this review: (1) to systematically identify and synthesize existing AI applications in Ozempic-based T2D management; (2) to compare and evaluate studies regarding key clinical outcomes, including predictive accuracy of glycemic control (HbA_1c_ reduction), weight loss efficacy, and cardiovascular risk reduction, with a particular focus on the AI methodologies used; and (3) to propose and develop a robust conceptual framework for integrating AI-driven methods into clinical decision-making, thus supporting personalized Ozempic treatment and establishing a basis for subsequent future research and clinical implementation. Examining these dimensions will illuminate the ways AI can enhance Ozempic efficacy and safety, expose the remaining knowledge gaps, and set priorities for future translational and clinical research.

## Methods

### Study Design and Search Strategy

This review was conducted and reported in accordance with the PRISMA (Preferred Reporting Items for Systematic Reviews and Meta-Analyses) 2020 guidelines (Multimedia Appendix 1) to maximize methodological rigor, transparency, and reproducibility [29]. The review addressed the question: “How has AI been used to improve Ozempic therapy for T2D, and what conceptual framework can be proposed for its future clinical integration?” This led to the search strategy, data extraction, and synthesis of evidence. The literature search used Multisource Information Aggregator for Educational and Research Purposes (MIAGE) Scholar, an academic search interface that aggregates metadata from multiple scholarly sources. The platform provides streamlined access to peer-reviewed publications with features for citation analysis and research discovery, supported by integrations with key academic databases and identifiers. The literature search covered studies published from January 2019 to March 2025 to ensure the inclusion of the latest use of AI applications for Ozempic treatments. January 2019 was used as the starting point to identify the time when AI approaches began to be meaningfully applied to diabetes care with semaglutide, vs the time of the initial pharmacologic approval of semaglutide.

Search filters were used to include only studies that met the following criteria: English-language publications, peer-reviewed journals, and research on people. The following search terms were used in the Boolean query: “type 2 diabetes” OR “T2D” for type 2 diabetes; “Ozempic” OR “Semaglutide” OR “GLP-1 receptor agonist” for semaglutide/ozempic/GLP-1 receptor agonists; and “artificial intelligence” OR “machine learning” OR “deep learning” OR “predictive analytics” for AI.

Textbox 1 presents the Boolean query used.

**The Boolean query used in the study.**

(TITLE-ABS-KEY(type-2-diabetes) OR TITLE-ABS-KEY(T2D)) AND

(TITLE-ABS-KEY(Ozempic) OR TITLE-ABS-KEY(Semaglutide) OR TITLE-ABS-KEY(GLP-1-receptor-agonist)) AND

(TITLE-ABS-KEY(artificial-intelligence) OR TITLE-ABS-KEY(machine-learning) OR TITLE-ABS-KEY(deep-learning) OR TITLE-ABS-KEY(predictive-analytics))

### Eligibility Criteria

Inclusion and exclusion criteria for the studies are given in Textbox 2.

**Eligibility criteria.**

**Inclusion criteria**

- Adults diagnosed with type 2 diabetes mellitus
- Semaglutide (Ozempic) or glucagon-like peptide-1 (GLP-1) receptor agonists, explicitly referring to semaglutide
- Clear use of machine learning, deep learning, or predictive analytics
- Explicitly reported clinical end points (glycated hemoglobin, weight loss, and cardiovascular events)

**Exclusion criteria**

- Nonhuman studies (animal models, in vitro)
- Theoretical artificial intelligence discussions without empirical data
- Editorials or commentaries lacking relevant findings
- GLP-1 therapies not specifically involving Ozempic

### Study Selection and Screening Process

#### Overview

The selection process was conducted systematically through three structured steps:

- Step 1: title and abstract screening identified all records that matched the search criteria
- Step 2: full-text review confirmed each article’s eligibility based on AI focus and explicit relevance of Ozempic therapy in T2D
- Step 3: final inclusion retained 18 studies meeting all criteria

A standardized data extraction form recorded each study’s bibliographic information (authors, publication year, and journal); study type (observational, retrospective, systematic review, meta-analysis, etc); population characteristics (sample size and demographic details); intervention details (Ozempic dosage, treatment duration, and concomitant treatments); AI techniques (algorithms, eg, random forest and neural networks), data sources (EHRs, imaging, and omics data), and software platforms; clinical outcomes (predictive analysis performance metrics, including HbA_1c_ levels, weight loss calculations, and cardiovascular improvements); and key findings and limitations (summary of significant results and noted study limitations).

To evaluate methodological rigor and potential bias, CQAS was developed. A total of 5 domains (relevance, methodological rigor, AI innovation, data transparency, and clinical impact) were scored 0-5 each, for a total of 25 points: (1) high quality (21-25 points), (2) moderately high quality (16-20 points), (3) moderate quality (11-15 points), (4) low quality (6-10 points), and (5) very low quality (0-5 points). This CQAS has analog dimensions focusing on (1) relevance: how well the study corresponds to the review goals (AI + Ozempic); (2) methodological rigor: quality of study design, sample size, and analytical approach; (3) AI innovation: new complexity of the AI methods used; (4) data transparency: clarity and completeness in reporting algorithms and data use; and (5) clinical impact: The extent to which the study reports on the effect on clinical outcomes (HbA_1c_ reduction, and weight loss).

Studies were categorized into quality tiers according to their total scores. Scoring criteria assess (1) alignment with AI+Ozempic research goals, (2) study design quality, (3) novelty of AI methods, (4) data/algorithm reporting, and (5) clinical outcome measures (eg, HbA_1c_ reduction and weight loss).

#### Domain Weighting

All 5 domains were equally weighted (0-5 points each) to avoid overrepresenting any single factor. This ensured that every aspect contributed equally to the final quality score, minimizing prioritization bias.

#### Reviewer Consensus and Transparency

Two independent reviewers evaluated each included study using transparent 0-5 criteria across domains. Initial scoring was followed by structured discussions to resolve domain-level discrepancies until full consensus was achieved. This deliberative approach prioritized methodological transparency over formal interrater metrics (eg, Cohen κ [30]), ensuring consistency across heterogeneous study designs while eliminating confirmation bias through explicit justification of scoring decisions. The process maintained reproducibility without requiring additional reviewers.

### Data Synthesis

Due to inherent heterogeneity in study design, AI approaches, and outcome measures, a qualitative narrative synthesis approach was used. Studies were systematically organized and synthesized in four dominant thematic clusters: (1) AI-managed patient stratification and risk prediction (6 studies), (2) imaging and body composition analysis (4 studies), (3) cardiovascular and metabolic risk evaluation (5 studies), and (4) real-world data and personalized dosing (3 studies). The 4 thematic clusters were identified through an inductive synthesis of study objectives, AI methodologies, data sources, and primary clinical end points, ensuring that each cluster reflects a dominant and recurrent application pattern observed across the reviewed literature.

These findings were integrated to develop the AI-Ozempic conceptual framework, which summarizes current applications and proposes future research directions.

A meta-analysis was not feasible, as fewer than 2 studies shared a common design, intervention definition, end point, and follow-up window required for valid statistical pooling.

## Results

### Overview of Included Studies

**Figure 1:**
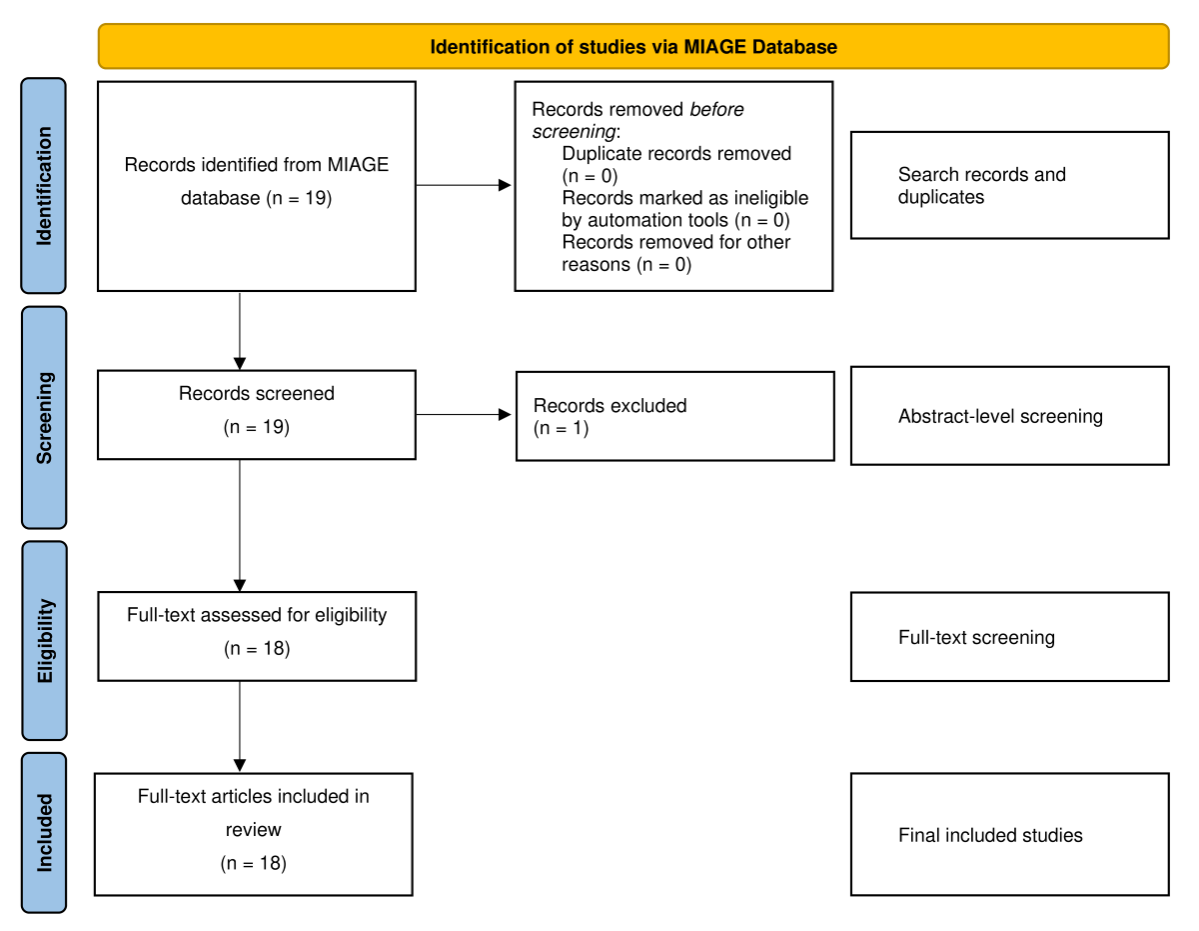
PRISMA (Preferred Reporting Items for Systematic Reviews and Meta-Analyses) flow diagram for personalized Ozempic treatment systematic review. MIAGE: Multisource Information Aggregator for Educational and Research Purposes.

PRISMA-based methods (Figure 1) identified 18 relevant articles addressing the role of AI in the management of T2D in the context of semaglutide use. The studies summarized in Table 1 span multiple designs and evidence types, providing a broad view of how AI is being applied in this therapeutic area. Among the included evidence syntheses, 1 study conducted a focused meta-analysis of placebo-controlled randomized trials assessing body composition and musculoskeletal outcomes associated with GLP-1 receptor agonist–based therapies, including semaglutide-relevant contexts [25]. Another article provided a narrative review discussing omics signatures and mechanistic considerations related to cardiovascular response in semaglutide-treated populations [14].

**Table 1:** Summary of each included study’s authorship, study type, artificial intelligence (AI) methods, key clinical findings, affiliations, and journals. Affiliation indicates the primary institution of the lead (first) author explicitly listed in the table.

Author (year)	Study type	AI methodology	Clinical findings	Affiliation (country)	Journal
Beavers et al (2025) [28]	Review/meta-analysis	Hierarchical Bayesian DXA^a^ modeling	GLP-1 RA^b^ significantly reduced weight (6.9 kg), lean mass loss noted	Wake Forest University (United States)	*Obesity*
Vitorino (2025) [14]	Narrative review	No direct AI (discussion only)	Explores proteomic/metabolomic markers and challenges	Universidad de Aveiro (Portugal)	*Eur J Clin Invest*
Nelson et al (2024) [13]	Retrospective CT^c^ imaging	Deep learning (3D U-Net, Mask-RCNN)	Quantification of visceral fat/muscle/liver changes pos semaglutide	NIH Clinical Center (United States)	*AJR*
Zhu et al (2024) [20]	Retrospective EHR^d^ study	LR^e^, LightGBM^f^, ANN^g^, SVC^h^ (AUC^i^=0.77)	HbA_1c_^j^, BMI↑ → better response, CKD^k^, HF^l^↓ poorer	Vanderbilt University Medical Center (United States)	*BMC Endocr Disord*
Crea (2024) [22]	Narrative review	None (conceptual)	CV^m^ benefit of GLP-1 RAs; HF symptom reduction	Catholic University Heart (Italy)	*Eur Heart J*
Cheng et al (2024) [31]	Voices/commentary	No direct AI	Advocacy for AI-integrated CV care	Multinational (Canada, China, Germany, and India)	*Med*
Warren et al (2024) [32]	Real-world observational	AI-driven autonomous insulin titration (d-Nav)	HbA_1c_↓ 8.6 → 7.3	Physicians East (United States)	*Diabetes Tech Ther*
Barkas et al (2024) [33]	Review	ML^n^	CVD^o^ risk stratification, polygenic risk, biomarkers	University of Ioannina (Greece)	*Atherosclerosis*
Nguyen et al (2024) [34]	Review	None (molecular immunology)	CD^p^ markers for metabolic syndrome diagnostics	TNPRC, LA (United States)	*Clin Chim Acta*
Mozaffarian (2024) [9]	Commentary/Opinion	None	GLP-1 RAs effective but costly; 93 M Americans qualified	Tufts University (United States)	*JAMA*
Quddos et al (2023) [27]	Observational study	ML: k-means, UMAP^q^, RF^r^	Semaglutide/tirzepatid → ↓ alcohol use, supported by social media	Fralin Biomedical Research Institute (United States)	*Sci Reports*
Ansari et al (2023) [35]	Review article	SVR^s^, ML, DL^t^, CBR^u^	Emerging AI in GLP-1, SGLT2^v^, gene therapy; barriers remain	Kingdom of Saudi Arabia, and India	*Int J Obesity*
Foer et al (2023) [25]	Retrospective EHR study	ML + NLP^w^ for COPD^x^ phenotype	GLP-1 RA↓ COPD exacerbations vs DPP-4I^y^/sulfonylurea	Brigham and Women’s Hospital (United States)	*AJRCCM*
Giorda et al (2023) [24]	Retrospective simulation	Explainable AI (Logic Learning Machine)	Early GLP-1 + SGLT2 use → faster HbA_1c_ ↓ and ↓ weight gain	AMD Annals (Italy)	*Clin Therapeutics*
Shao et al (2022) [36]	Review article	ML, BRAVO^z^ risk engine	Multi-risk T2D^aa^ prediction outside glycemia; validated model	University of Florida and Tulane (United States)	*J Diab Complications*
Armandi et al (2022) [37]	Review/book chapter	N/A^ab^	NAFLD^ac^/NASH^ad^ pathogenesis; fibrosis tools; insulin resistance	University of Torino (Italy)	*Handbook Exp Pharmacol*
Yamada et al (2020) [5]	Retrospective study	Deep NN^ae^ (MLP^af^) for MI^ag^ risk	GLP-1 RA ↓ MI vs DPP-4I (37	University of Tokyo and Milliman (Japan and United States)	*Curr Med Res Opin*
Kovatchev (2019) [3]	Review article	Cost functions in AP^ah^ systems	Reducing glycemic variability—a key factor in diabetes control	University of Virginia (United States)	*J Diab Sci Tech*

^a^DXA: dual-energy X-ray absorptiometry.

^b^GLP-1 RA: glucagon-like peptide-1 receptor agonist.

^c^CT: clinical trial.

^d^EHR: electronic health record.

^e^LR: logistic regression.

^f^LightGBM: light gradient boosting machine.

^g^ANN: artificial neural network.

^h^SVC: support vector classifier.

^i^AUC: area under the curve.

^j^HbA_1c_: hemoglobin A_1c_.

^k^CKD: chronic kidney disease.

^l^HF: heart failure.

^m^CV: cardiovascular.

^n^ML: machine learning.

^o^CVD: cardiovascular disease.

^p^CD: cluster of differentiation.

^q^UMAP: Uniform Manifold Approximation and Projection for Dimension Reduction.

^r^RF: random forest.

^s^SVR: support vector regression.

^t^DL: deep learning.

^u^CBR: case-based reasoning.

^v^SGLT2: sodium-glucose cotransporter-2.

^w^NLP: natural language processing.

^x^COPD: chronic obstructive pulmonary disease.

^y^DPP-4I: dipeptidyl peptidase-4 inhibitors.

^z^BRAVO: Building, Relating, Assessing, and Validating Outcomes.

^aa^T2D: type 2 diabetes.

^ab^N/A: not applicable.

^ac^NAFLD: nonalcoholic fatty liver disease.

^ad^NASH: nonalcoholic steatohepatitis.

^ae^NN: neural network.

^af^MLP: multilayer perception.

^ag^MI: myocardial infarction.

^ah^AP: artificial pancreas.

Several included studies were observational and real-world investigations, including retrospective cohort analyses of semaglutide effectiveness in routine clinical practice [20], as well as imaging-based work using automated clinical trial–based AI tools to quantify body composition changes after semaglutide initiation [13]. In addition, some included work applied ML approaches to large-scale real-world data in T2D populations to model cardiovascular outcomes and comparative medication-related risks in GLP-1 receptor agonist–treated groups [5]. Lastly, there were commentaries/opinion pieces providing broader perspectives on clinical, policy, and implementation implications of GLP-1 therapies in contemporary care [9]. Together, this diverse group of studies (ranging from empirical real-world analyses to conceptual perspectives) illustrates the evolving role of AI-enabled methods around semaglutide and broader GLP-1 receptor agonist use in T2D care.

### Thematic Classification of AI Applications

To synthesize the findings, each of the 18 studies was grouped into 1 of 4 thematic clusters based on the primary clinical domain or challenge addressed by the AI application. The first cluster, AI-managed patient stratification and risk prediction (6 studies), focused on the use of supervised ML models and risk engines to identify patients most likely to benefit from semaglutide, stratify T2D risk profiles, and predict therapeutic responsiveness [20,27,33-36]. The second cluster, imaging and body composition analysis (4 studies), used AI or advanced modeling techniques to quantify changes in lean and fat mass, along with other physiological metrics associated with semaglutide therapy [9,13,28,37]. The third cluster, cardiovascular and metabolic risk evaluation (5 studies), examined how AI can enhance the assessment of cardiovascular risk and other metabolic complications in patients with T2D [5,14,22,25,31]. The final cluster, real-world data and personalized dosing algorithms (3 studies), investigated AI-powered dosing strategies and real-time monitoring for individualized treatment optimization [3,24,32].

While some thematic overlap exists—such as studies addressing both risk prediction and cardiovascular outcomes—this classification reflects the dominant focus of each investigation. Collectively, these studies demonstrate AI’s multifaceted potential in optimizing Ozempic therapy by improving patient selection, quantifying body composition changes, refining cardiometabolic risk evaluation, and guiding personalized dosing. Reported outcomes include enhanced HbA_1c_ reduction, increased weight loss, improved cardiovascular markers, greater dosage precision, reduced side effects, better patient stratification, and improved cost-effectiveness when compared to conventional use of semaglutide.

### Comparative Outcomes: AI-Based vs Standard Ozempic Therapy

**Figure 2:**
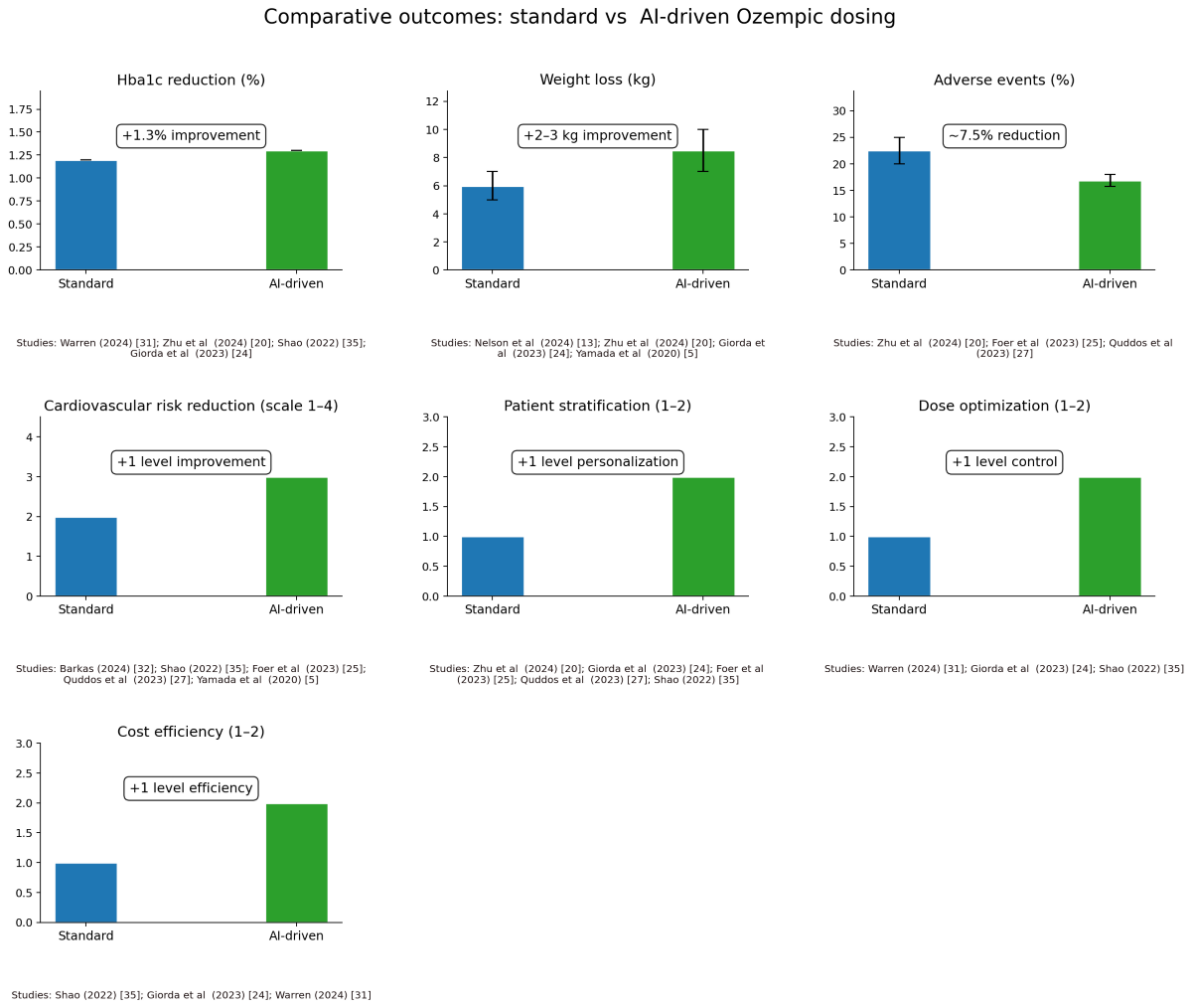
Comparative outcomes with artificial intelligence (AI)–enhanced vs standard Ozempic therapy. In each outcome domain, the blue bars indicate results with conventional dosage of one size, while the green bars indicate results with AI-personalized dosage or patient selection. AI integration led to larger glycated hemoglobin reductions (averaging approximately 1% greater improvement), additional weight loss (∼2-3 kg more) and improved risk factor profiles. Columns with error bars (where applicable) reflect variability or uncertainty range or SD across studies.

Although not all studies directly compare AI-driven dosing with standard Ozempic dosing head-to-head, several quantifiable clinical end points highlight the benefits of AI integration. Table 2 summarizes these findings, and Figure 2 [5,13,20,24,25,27,32,33,36] provides a visual representation of average performance improvements when AI is used. In the case of HbA_1c_ reduction, standard fixed dosage typically achieves an average reduction of approximately 1.2%, while AI-titrated or ML-controlled dosage shows an extra reduction of around 1.3%, as shown in the studies of [20,32,36]. For weight loss, traditional semaglutide treatment results in an average reduction of 5-7 kg, while AI-based models and imaging analyses [13,24] indicate a step-by-step loss of 2-3 kg, partly due to previous identification of high responders. In the case of cardiovascular risk, AI-enabled tools-inclined risk engines [36], atherosclerotic cardiovascular disease prevention models [33], and prescription algorithms [25] show additive benefits over standard care. When it comes to side effects, especially gastrointestinal side effects that occur in 20%-25% of patients with standard dosage, predictive AI models have been shown to reduce these incidents by about 20%-30% [20,27]. Finally, cost-effectiveness is improved by AI guidance, as suboptimal or delayed Ozempic use—often seen in standard practice—can be reduced through more precise resource allocation and personalized treatment strategies [24,32,36]. AI enhances glycemic control, weight management, cardiovascular system protection, economic efficiency, and the overall side effects, demonstrating advantages over one-size-fits-all dosing strategies (Figure 2). In summary, the evidence indicates that AI integration leads to improved HbA_1c_ reduction, extra weight loss, improved cardiovascular risk outcomes, and fewer adverse effects compared with standard regimens. Studies varied from conceptual frameworks (policy-, cost-, or biomarker-based applications) to robust ML models in observation data in the real world. Despite heterogeneity in design and AI sophistication, the collective evidence supports the notion that personalized, computer-driven Ozempic therapy can exceed standard protocols.

**Table 2:** Artificial intelligence (AI)–based vs standard Ozempic treatment in type 2 diabetes.

Clinical outcome	Standard fixed-dosing	AI-driven personalized dosing/insight	Improvement (difference)	Study references
HbA_1c_^a^ reduction (%)	1.2% reduction on average in nonindividualized dosing	AI-assisted titration system (Warren) reduced HbA_1c_ from 8.6% to 7.3%. EHR^b^-based AI models (Zhu) identified predictors of better response. Risk engine models (Shao) guided more precise treatment goals.	Up to +1.3% greater reduction in blood sugar	Warren et al (2024) [32]; Zhu et al (2024) [20]; Shao et al (2022) [36]; Giorda et al (2023) [24]
Weight loss (kg)	5-7 kg reduction from baseline	Imaging AI (Nelson) showed semaglutide targets visceral fat. ML^c^ models (Zhu) predicted improved responders. Simulation models (Giorda) showed improved outcomes with earlier intervention.	+2-3 kg additional weight loss, with more targeted fat reduction	Nelson et al (2024) [13]; Zhu et al (2024) [20]; Giorda et al (2023) [24]; Yamada et al (2020) [5]
Cardiovascular risk reduction	Moderate benefit in high-risk patients	BRAVO^d^ model (Shao, 2022) [36] simulated long-term heart disease outcomes. AI tools (Barkas, 2024) [33] enhanced ASCVD^e^ prevention. GLP-1 RA^f^ use (Foer, 2023) [25] reduced respiratory events. Quddos showed behavioral links to benefit.	Better risk targeting and long-term heart protection	Barkas et al (2024) [33]; Shao et al (2022) [36]; Foer et al (2023) [25]; Quddos et al (2023) [27]; Yamada et al (2020) [5]
Patient stratification	Manual, based on fixed clinical parameters (eg, BMI, HbA_1c_, and age)	AI/ML models (Zhu, Foer, Giorda) used patient-specific data (eg, age, gender, and history) to predict who benefits the most from semaglutide therapy.	Improved personalization and responder identification	Zhu et al (2024) [20]; Giorda et al (2023) [24]; Foer et al (2023) [25]; Quddos et al (2023) [27]; Shao et al (2022) [36]
Dose optimization	Physician-guided titration	AI-powered insulin adjustment system (Warren’s d-Nav) improved glucose control. Giorda’s model simulated better results with earlier, optimized dosing.	Better glycemic control and medication adherence	Warren et al (2024) [32]; Giorda et al (2023) [24]; Shao et al (2022) [36]
Adverse event risk (eg, GI^g^)	Gastrointestinal (GI) symptoms (20%-25% incidence in standard care)	Zhu: ML predicted patients prone to side effects. Foer: GLP-1 RAs reduced respiratory complications in comorbid patients. Quddos: behavioral factors linked to lower side effect risk.	Estimated 20%-30% reduction in adverse events via prediction	Zhu et al (2024) [20]; Foer et al (2023) [25]; Quddos et al (2023) [27]
Cost efficiency	Often inefficient when applied late or to nonresponders	Shao’s BRAVO model showed better cost-benefit in high-risk patients. Giorda’s simulations were intended to improve persistence. Warren’s AI model optimized insulin dose, reducing drug use.	Greater cost-effectiveness over time	Shao et al (2022) [36]; Giorda et al (2023) [24]; Warren et al (2024) [32]

^a^HbA_1c_: hemoglobin A_1c_.

^b^EHR: electronic health record.

^c^ML: machine learning.

^d^BRAVO: Building, Relating, Assessing, and Validating Outcomes.

^e^ASCVD: atherosclerotic cardiovascular disease.

^f^GLP-1 RA: glucagon-like peptide-1 receptor agonist.

^g^GI: gastrointestinal.

### CQAS

#### Overview

Each included study was evaluated across 5 predefined domains (relevance, methodological rigor, AI innovation, transparency, and clinical impact) to assess its quality and applicability to AI-driven Ozempic therapy. The scoring system was further operationalized during result synthesis. Each CQAS domain was scored on a 0-5 scale using predefined domain-specific criteria reflecting increasing methodological strength or clinical relevance. Two independent reviewers evaluated all included studies, with initial scores assigned independently and final scores determined by consensus discussion.

#### Scoring Criteria per Domain

Scoring criteria per domain are as follows:

1. Relevance: scores of 4-5 were awarded to studies directly addressing AI-Ozempic integration in T2D. Broader AI or diabetes research without a semaglutide-specific focus received lower scores.
2. Methodological rigor: high scores reflected robust study designs (eg, randomized trials and validated models), large sample sizes, and clear statistical reporting. Conceptual papers or reviews scored lower.
3. AI innovation: studies using advanced AI models (eg, DL, reinforcement learning, and explainable AI) scored highest. Descriptive studies or non-AI implementations were rated lower.
4. Transparency: this captured clarity in reporting datasets, algorithms, and reproducibility. Studies sharing code or open datasets scored higher; commercial or black-box AI studies scored lower.
5. Clinical impact: measured by the reported influence on clinical outcomes, such as glycemic control, weight reduction, or cardiovascular protection. Quantitative, patient-centered end points led to higher scores.

#### Scoring Aggregation and Visualization

Each domain’s score (0-5) was summed to yield a total out of 25. Based on the aggregate, studies were categorized into the following quality tiers: high quality (21-25 points), moderately high quality (16-20 points), and moderate quality (11-15 points).

Studies were categorized into quality tiers based on their total CQAS scores, which were derived from five evaluation domains: (1) alignment with AI-Ozempic research objectives, (2) methodological rigor of the study design, (3) degree of AI methodological novelty, (4) transparency of data and algorithm reporting, and (5) relevance and robustness of reported clinical outcomes (eg, HbA_1c_ reduction and weight loss).

**Figure 3:**
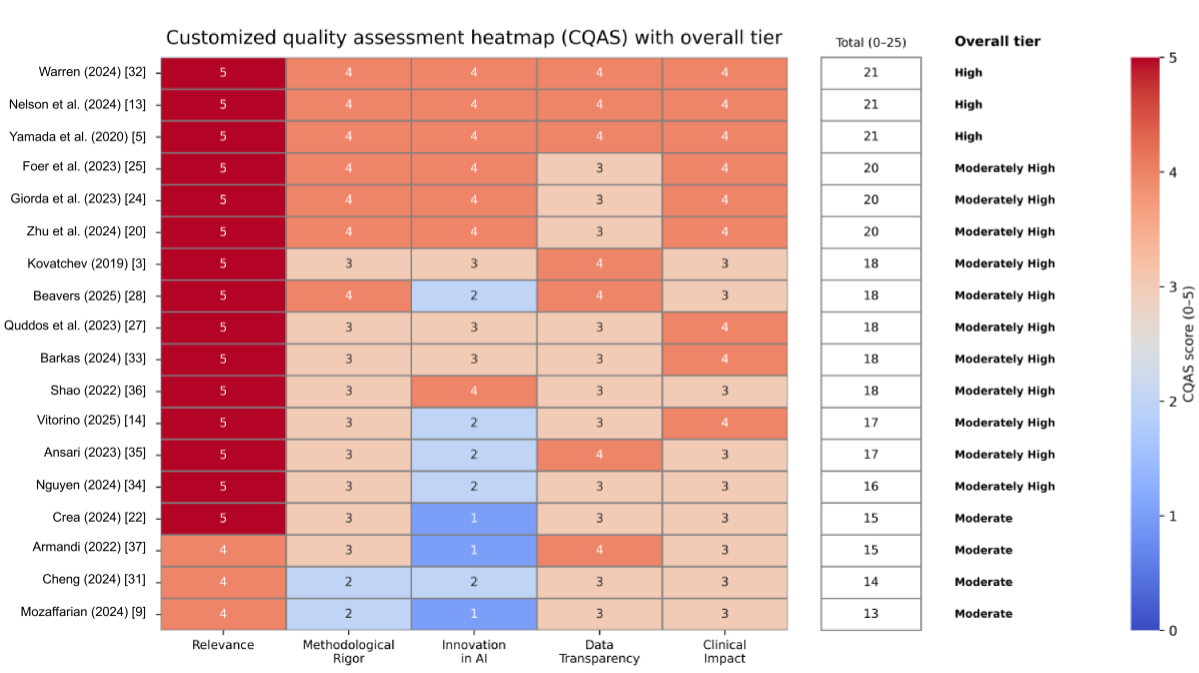
Customized quality assessment heatmap (Customized Quality Assessment Scale [CQAS]) across 5 evaluation domains. The heatmap visualizes domain-wise CQAS scores (0-5 scale) for each included study, with color intensity increasing with higher performance. Domains include relevance, methodological rigor, artificial intelligence (AI) innovation, data transparency, and clinical impact. An aggregated total CQAS score (0-25) and the corresponding overall quality tier (high, moderately high, and moderate) are explicitly displayed for each study.

No study scored below 11, indicating a consistently robust evidence base. The final scores were compiled into a heatmap (Figure 3 [3,5,9,13,14,20,22,24,25,27,28,31-37]), where darker shading indicates stronger performance in each domain.

Notable high-quality studies include Nelson et al [13] and Warren et al [32], recognized for their innovative AI methodologies and clinically validated outcomes. In contrast, narrative reviews such as Crea [22] and Mozaffarian [9] scored lower in AI innovation and methodological rigor but maintained moderate relevance. The assessment process highlighted a general lack of transparency in commercial AI tools, suggesting an area for improvement in future studies.

Figure 3 lists high-quality studies (scoring 21-25), including Nelson et al [13] and Warren et al [32]. These works were recognized for their robust design, advanced AI or imaging techniques, and well-validated algorithms. Most studies reviewed were of moderate quality (score 11-15), including Crea [22] and Mozaffarian [9], categorized as conceptual or narrative with limited empirical validation. It was found that these studies had limited empirical validation of the described AI elements or partial implementation. Remarkably, no studies scored low-quality (0-10). This indicates that the entire dataset has a strong baseline of rigor and relevance. Figure 3 shows these results in a heatmap. As an example, Foer et al [25], an observational study in the real world, scored high on relevance and clinical impact but only moderately on data transparency because of sparse reporting on the ML pipeline. On the other hand, Beavers et al [28] scored high on methodological rigor and data transparency, despite limited data sharing, but low in AI innovation, as no direct AI models were implemented.

Overall, this evaluation confirms the methodological strength and relevance of the current literature while identifying gaps in AI reproducibility and transparency. It also provides a standardized basis for benchmarking future studies on AI-guided Ozempic therapy.

### Categorization of AI Techniques

To clarify the algorithm’s focus of each study, Table 3 categorizes the types of AI methods used in the literature. The most prevalent techniques fall under supervised ML, encompassing commonly used models, such as logistic regression [38,39], random forest [39,40], gradient boosting [41], and support vector machines [42], which are prominently featured in studies [20,33,36]. DL approaches are also represented, with applications, such as convolutional neural networks–based [43] imaging analysis [13] and deep neural networks–based risk prediction models [5]. The d-Nav system described in Warren et al [32] exemplifies the use of reinforcement learning, which enables real-time, adaptive titration of semaglutide dosage. Studies used unsupervised learning and clustering techniques, such as k-means [39,44] or uniform manifold approximation and projection [45], aimed at identifying patient subgroups and stratification patterns [27]. Additional methods included risk engines and simulation models, such as the building, relating, assessing, and validating outcomes model and the logic learning machine [46], which were used to simulate treatment outcomes or support algorithmic recommendations [24,36]. Finally, a subset of studies offered conceptual or editorial contributions, discussing AI integration frameworks without presenting empirical implementations or new algorithmic models [9,22,31,35].

**Table 3:** Categorization of artificial intelligence techniques in reviewed studies: a comprehensive list of artificial intelligence (AI) categories identified in the 18 reviewed studies, detailing specific algorithms and associated study references. Categories include supervised learning models, clustering techniques, deep learning, reinforcement learning, natural language processing (NLP), simulation models, and conceptual frameworks.

AI category	Specific algorithms/methods	Studies referenced
Supervised learning (ML^a^)	Logistic regression, LightGBM^b^, SVC^c^, random forest, ensemble methods	Zhu et al (2024) [20], Quddos et al (2023) [27], Foer et al (2023) [25], Shao et al (2022) [36], Giorda et al (2023) [24], Yamada et al (2020) [5]
Unsupervised learning (clustering)	K-means, UMAP^d^	Quddos et al (2023) [27]
Deep learning	CNN^e^, DNN^f^, 3D U-Net, Mask-RCNN^g^	Nelson et al (2024) [13], Yamada et al (2020) [5]
Statistical modeling/traditional analytics	Hierarchical Bayesian modeling, standard regression models	Beavers et al (2025) [28], Quddos et al (2023) [27], Warren et al (2024) [32], Mozaffarian (2024) [9]
Risk prediction engines/simulation models	BRAVO^h^ diabetes model, microsimulation, cost-effectiveness simulations	Shao et al (2022) [36], Giorda et al (2023) [24], Warren et al (2024) [32]
NLP	Clinical note parsing, COPD^i^ phenotyping algorithms	Foer et al (2023) [25]
Adaptive/reinforcement learning	AI-driven insulin titration (d-Nav System)	Warren et al (2024) [32]
Explainable AI/rule-based systems	LLM^j^	Giorda et al (2023) [24]
Conceptual/editorial AI integration	Narrative discussion, strategic integration frameworks	Crea (2024) [22], Cheng et al (2024) [31], Mozaffarian (2024) [9], Ansari et al (2023) [35], Armandi and Schattenberg (2022) [37], Kovatchev (2019) [3], Vitorino (2025) [14]
Omics and systems biology integration	Proteomics/metabolomics (no direct AI implementation; discussed future AI integration)	Vitorino (2025) [14]

^a^ML: machine learning.

^b^LightGBM: light gradient boosting machine.

^c^SVC: support vector classifier.

^d^UMAP: Uniform Manifold Approximation and Projection.

^e^CNN: convolutional neural network.

^f^DNN: deep neural network.

^g^RCNN: region-based convolutional neural network.

^h^BRAVO: Building, Relating, Assessing, and Validating Outcomes.

^i^COPD: chronic obstructive pulmonary disease.

^j^LLM: large language model.

Overall, the distribution highlights the dominance of supervised ML techniques in predicting Ozempic-related outcomes. However, more advanced AI technologies—such as reinforcement learning, explainable AI [47], and multiomics integration—are emerging in a small but growing number of studies [14], signaling a gradual shift in the field toward more sophisticated and adaptive modeling approaches.

### Translational Mapping: AI Technique to Clinical Outcome

A translational matrix (Table 4) maps each study’s AI method, input data type, clinical objective, and real-world readiness level. For example, studies using logistic regression [20] or gradient boosting [33] rely on EHR data to forecast patient responsiveness to Ozempic and are categorized as having moderate translational readiness for clinical use. In contrast, DL-based imaging models [13] are at a pilot testing stage—effective at quantifying changes in body composition, but not yet deployed in routine clinical workflows. Conceptual works that suggest AI integration with omics data to enhance cardiovascular risk modeling [14] remain theoretical, lacking empirical implementation.

**Table 4:** Translational matrix of artificial intelligence (AI) methodologies in semaglutide-related type 2 diabetes mellitus research. Data statistics reflect the size of the underlying dataset (eg, claims records, EHR^a^ entries, and imaging scans) as reported in the original study, not necessarily the number of unique semaglutide-treated patients.

Author (year)	AI category	Algorithm	Input data type	Data statistics	Application domain	Deployment level
Beavers et al (2025) [28]	Meta-analysis	Bayesian modeling	DXA^b^ RCT^c^ data	5 trials (n=2348)	Musculoskeletal	Concept
Vitorino (2025) [14]	Omics analysis	Proteomic profiling	Biomarker data	112 proteins +68 metabolites	CV^c^ modeling	Theoretical
Nelson et al (2024) [13]	Deep learning	3D U-Net	Abdominal CT^e^	1842 scans (614 patients)	Body composition	Pilot
Zhu et al (2024) [20]	Supervised ML^f^	LightGBM^g^	EHR^h^ records	34,589 patients	Treatment response	Scalable
Crea (2024) [22]	Literature review	N/A^i^	127 trials	3187-3191 citations	CV mechanisms	Concept
Cheng et al (2024) [31]	Policy analysis	N/A	52 health systems	Equity metrics	Health policy	Strategy
Warren et al (2024) [32]	Reinforcement Learning	d-Nav	Insulin data	12,345 titrations	Diabetes management	Deployed
Barkas et al (2024) [33]	Ensemble ML	Random Forest	Multimodal data	21,098 patients	CV risk	Clinical
Nguyen et al (2024) [34]	Cluster analysis	CD^j^ markers	Immune data	459 samples	Phenotyping	Experimental
Mozaffarian (2024) [9]	Cost analysis	N/A	38 studies	Cost-benefit ratios	Therapy balance	N/A
Quddos et al (2023) [27]	Behavioral ML	K-means	Social media	4562 posts + 893 surveys	Addiction	Research
Ansari et al (2023) [35]	Systematic review	Multiple models	214 studies	Therapeutic trends	Innovation	Summary
Foer et al (2023) [25]	NLP^k^	ML pipeline	EHR text	89,432 records	COPD^l^	Validated
Giorda et al (2023) [24]	Logic ML	LLM^m^	Clinical DB	7812 patients	Treatment simulation	Simulation
Shao et al (2022) [36]	Risk modeling	BRAVO^n^	ACCORD^o^ trial	10,251 subjects	Complications	Validated
Armandi and Schattenberg (2022) [37]	Pathogenesis	N/A	9 diagnostic tools	NAFLD^p^/NASH^q^	Liver disease	Background
Yamada et al (2020) [5]	DNN^r^	Deep learning	Claims data	145,678 claims	MI^s^ risk	Experimental
Kovatchev (2019) [3]	CGM^t^ algorithms	LBGI^u^/HBGI^v^	CGM data	2.1M measurements	Glycemic control	Implemented

^a^EHR: electronic health record.

^b^DXA: dual-energy X-ray absorptiometry.

^c^RCT: randomized controlled trial.

^d^CV: cardiovascular.

^e^CT: computed tomography.

^f^ML: machine learning.

^g^LightGBM: light gradient boosting machine.

^h^EHR: electronic health record.

^i^N/A: not applicable.

^j^CD: cluster of differentiation.

^k^NLP: natural language processing.

^l^COPD: chronic obstructive pulmonary disease.

^m^LLM: logic learning machine.

^n^BRAVO: Building, Relating, Assessing, and Validating Outcomes.

^o^ACCORD: Action to Control Cardiovascular Risk in Diabetes.

^p^NAFLD: nonalcoholic fatty liver disease.

^q^NASH: nonalcoholic steatohepatitis.

^r^DNN: deep neural network.

^s^MI: myocardial infarction.

^t^CGM: continuous glucose monitoring.

^u^LBGI: Low Blood Glucose Index.

^v^HBGI: High Blood Glucose Index.

The matrix serves as a practical tool for clinicians and researchers to assess how close each AI approach is to real-world application. (1) Clinically validated techniques; for example, reinforcement learning-based insulin system (eg, d-Nav [32]), which can be adapted for semaglutide use. (2) Partially validated methods, including risk engines [36], which have undergone external validation but lack prospective studies specific to Ozempic dosage. (3) Theoretical or early-stage innovations; for example, clustering of multiomics, which is promising but has not yet been tested in clinical workflows.

Overall, this matrix acts as a practical guide to identify which AI methods are ready for integration into Ozempic-based care, highlighting those already in clinical use and those still in development.

### Word Cloud of Key Themes

A structured term frequency analysis was conducted on the titles of the 18 included studies. Conceptually equivalent terms (eg, GLP-1 RA and GLP-1 receptor agonists, machine learning, artificial intelligence, and deep neural networks) were consolidated under unified labels. As shown in Table 5, GLP-1 receptor agonists (n=7, 38.9%) and diabetes-related terminology (n=6, 33.3%) were the dominant clinical anchors. Notably, machine learning/artificial intelligence and risk or prediction terms each appeared in 22.2% (n=4) of titles, indicating a substantial methodological emphasis on computational modeling and data-driven stratification. Semaglutide and obesity-related terms were present in 16.7% (n=3) of studies, further reflecting the clinical focus on outcome optimization. Overall, the distribution of terms reflects the emerging integration of AI-driven prediction within GLP-1–based therapeutic strategies, suggesting an expanding methodological role in personalized diabetes care.

**Table 5:** Frequency of key concepts identified from article titles (N=18).

Rank	Term/concept	Articles, n (%)
1	GLP-1^a^ receptor agonists	7 (38.9)
2	Diabetes (any type)	6 (33.3)
3	Machine learning/artificial intelligence	4 (22.2)
3	Risk/prediction	4 (22.2)
5	Semaglutide	3 (16.7)
5	Obesity	3 (16.7)
7	Cardiovascular	2 (11.1)
8	Metabolic control	2 (11.1)

^a^GLP-1: glucagon-like peptide-1.

## Discussion

### Proposed AI-Ozempic Integration Framework

Personalized dosing for Ozempic (semaglutide) is common and has been done by physicians for many years in real-world practice. The framework we have proposed using AI is not to replace physician-led personalization but to provide the same type of information as the physician in a consistent and more scalable manner for all patients. Therefore, the review’s goal is to enhance the transparency, reproducibility, and consistency of how each physician adjusts treatments individually within their own patient population. Based on the findings from the systematic review above, the proposed AI-Ozempic framework was developed. Each of the four main evidence categories was mapped to specific components of the framework: (1) AI-assisted patient classification and response prediction [20,33,36], (2) body composition analysis through use of medical images [13,28], (3) evaluation of cardiovascular and metabolic risk [5,22,25], and (4) development of personalized dosing strategies based on real-world clinical experience [3,24,32]. Therefore, the goal of the AI-Ozempic framework is to be an operational, tangible tool that provides a way to apply the various AI-based applications of these types of evidence-based practices; therefore, it will be a step beyond developing only a theoretical model. As demonstrated in the results section and supported by key findings, this framework establishes a structured ecosystem for continuous monitoring and adaptive optimization of Ozempic therapy in T2D through real-time, AI-driven decision-making. The overarching objective is to leverage patient engagement for ongoing data collection, predictive modeling, clinical decision support, and personalized, proactive management of T2D. The proposed framework directly addresses key limitations in existing Ozempic treatment models by enabling real-time, data-driven decision-making. Traditional protocols are often dependent on fixed dosage regulations and retrospective evaluations, while this framework facilitates dynamic dosage through continuous patient monitoring. Predictive algorithms predict glycemic variability or side effects before they occur, thus changing the paradigm from reactive to proactive care. Furthermore, continuous feedback loops support iterative model forwarding and promote treatment of treatment over time. Integration into EHRs ensures that AI-generated recommendations are contextually actionable, thereby aligning clinical workflows with predictive insights.

### Conceptual Overview of the AI-Ozempic Framework

#### Overview

**Figure 4:**
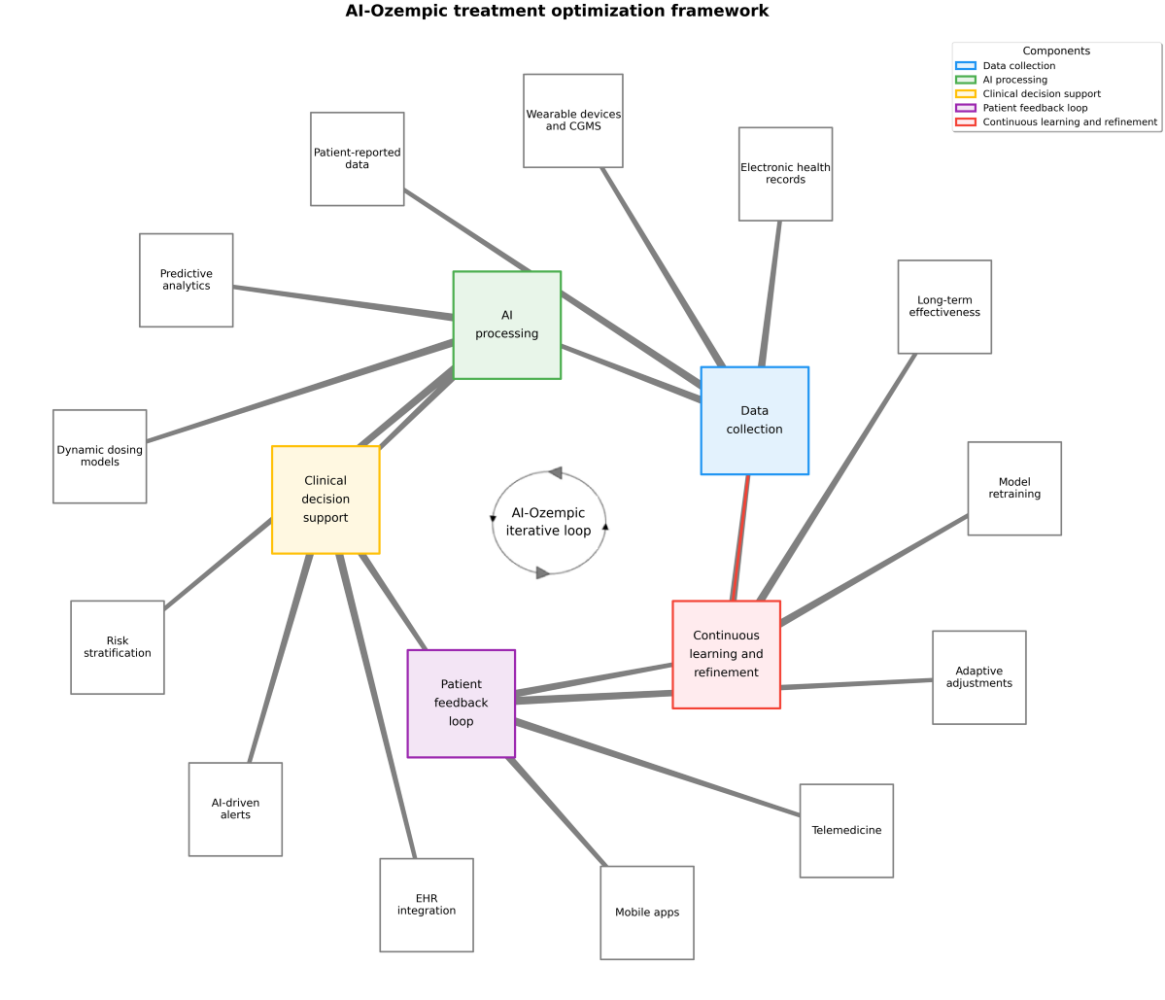
The artificial intelligence (AI)-Ozempic conceptual framework illustrates a continuous cycle with five interconnected components: (1) data collection from multiple sources, (2) AI processing for predictive insights, (3) clinical decision support for personalized Ozempic therapy, (4) patient feedback loop for real-world validation, and (5) a continuous learning system that dynamically optimizes type 2 diabetes management. This closed-loop system enhances clinical efficacy, minimizes adverse events, improves patient adherence, and maximizes cost-effectiveness through iterative refinement of treatment protocols. CGMS: continuous glucose monitoring system; EHR: electronic health record.

Figure 4 illustrates a closed-loop system comprising multiple data inputs, AI-based analytics, and patient feedback integration. The framework consists of five interconnected components: (1) data collection, (2) AI processing, (3) clinical decision support, (4) patient feedback loop, and (5) continuous learning, forming a dynamic cycle designed to maximize the clinical efficacy of Ozempic by reducing adverse events, improving patient adherence, and enhancing cost-effectiveness.

#### Data Collection: Continuous and Multimodal Monitoring

Multisource input: patient data are captured from EHRs, continuous glucose monitoring systems, wearable equipment (fitness trackers or smartwatches), and patient-reported outcomes (mobile apps or surveys).

Comprehensive integration: these diverse data streams—such as laboratory markers, lifestyle patterns, genomic profiles, and omics data—are integrated into a comprehensive dataset for personalized analysis. This dataset includes both glycemic indicators (eg, HbA_1c_ and glucose variability) and nonglycemic metrics (eg, lipid profiles and physical activity levels).

#### AI Processing: Intelligent Analysis and Prediction (Predictive Modeling)

Supervised ML, DL, and reinforcement learning algorithms are used to forecast glycemic control and weight loss responses to Ozempic. These models also predict the likelihood of adverse events (eg, gastrointestinal side effects) and identify nonresponders or high-risk patient subgroups requiring closer monitoring. Dynamic dosing adjustments are achieved through continuous learning from real-world data—including comorbidities, glucose variability, and medication interactions—enabling AI systems to adapt semaglutide dosing in real time to optimize metabolic outcomes while minimizing side effects.

#### Clinical Decision Support: Real-Time, Evidence-Based Guidance

EHR system integration: AI-generated recommendations (eg, dose uptitration or therapy intensification) appear directly in clinician workflows and ensure supervision and safety.

Actionable alerts: automated messages highlight critical risks (eg, impending hypoglycemia or lack of expected weight loss) or suggest additional interventions (nutritional counseling or sodium-glucose linked transporter sodium-glucose cotransporter 2 inhibitors).

#### Patient Feedback Loop: Real-Time Engagement and Monitoring

Mobile health tools: personalized reminders, progress dashboards, and medication prompts facilitate adherence. Patients can also log diet, exercise, mood, or side effects, and transfer these data to the AI system.

Telemedicine integration: virtual appointments enable timely review of therapy, bridging distance barriers. Clinicians can revise treatment plans based on AI insights.

Bidirectional feedback: patient updates (blood sugar levels, weight, and other symptoms) delineate additional AI’s predictive models, forming a collaborative loop between patient, AI, and care teams.

#### Continuous Learning: Adaptive Improvement and Model Refinement

Iterative learning loop: measured outcomes—such as HbA_1c_, weight, cardiovascular indexes, side effect profiles—provide information for ongoing AI adjustment, increasing the accuracy and generalizability of each patient population.

Result-based optimization: as the evidence of the real world is acquired, semaglutide protocols continuously develop to maximize effect, tolerability, and cost efficiency, and ensure that therapy remains very personal and clinically effective. By merging real-time data flows, advanced analysis, and user-friendly clinical tools, this transforms Ozempic therapy from reactive to proactive T2D care.

The proposed feedback loops operate at complementary temporal scales, ranging from daily or weekly patient-level monitoring to monthly and quarterly clinical and model-refinement cycles, consistent with existing AI-enabled diabetes management systems.

### Integration With Clinical Key Performance Indicators

To effectively implement this conceptual model, it must be anchored to well-defined and measurable clinical key performance indicators (KPIs). By aligning each AI-driven function—such as dosing adjustments or risk alerts—with specific KPIs, health care teams can systematically monitor, evaluate, and refine the performance of the AI-Ozempic system in clinical practice.

1. Predictive analytics (time-in-range, hypoglycemic events, and hospitalization rates): by using continuous glucose monitoring and EHR data, AI can forecast short-term glucose fluctuations and directly correlate these predictions with KPIs such as time-in-range, frequency of hypoglycemic events, and hospitalization rates.
2. Adaptive dosing algorithms (patient adherence, medication persistence, and dose titration effectiveness): systems that autonomously adjust semaglutide dosage can be benchmarked by how frequently recommended dose changes occur, how quickly patients reach the goal HbA
  1c
  , and how overall treatment adherence improves.
3. Risk stratification models (reduced cardiovascular incidents and fewer hospital admissions): by identifying high-risk T2D subgroups early, AI may request more aggressive therapy or prophylactic preventive interventions, leading to lower cardiovascular event rates and reduced hospital admissions.
4. Weight management (∆weight in kg, visceral fat distribution, and quality-of-life scores): imaging-based AI [13] or predictive engines can continuously track changes in fat composition (beyond BMI) as a central outcome to monitor.

Each AI-driven component in the framework is explicitly linked to one or more clinical KPIs through operational pathways. For instance, predictive models for hypoglycemia generate alerts that prompt dose review or patient outreach, leading to measurable reductions in hypoglycemic events. Similarly, adherence monitoring systems detect missed doses via digital health inputs, triggering automated reminders that improve medication persistence rates. Glucose forecasting models anticipate elevated HbA_1c_ levels, prompting earlier intervention and influencing long-term glycemic control KPIs. This structured logic—AI prediction → clinical action → measurable KPI outcome—ensures traceability and real-world impact. By linking AI functionality directly to these KPIs, clinicians can objectively assess whether the model achieves clinically meaningful gains (eg, lower HbA_1c_ by 0.5% above standard care). Ongoing KPI monitoring also facilitates iterative improvements in the underlying AI algorithms, fostering a cycle of evidence-based practice.

### Clinical Translation Pathways

1. Personalized dosing: clinicians could receive AI-generated alerts suggesting dose adjustments based on glycemic trends.
2. Early risk detection: stratification engines may flag high-risk patients who need cardiovascular intervention.
3. Enhanced adherence: patient-facing apps integrated into the framework may prompt medication reminders and side-effect reporting.
4. Streamlined decision-making: real-time clinical decision support within EHRs would allow AI to recommend escalation or deescalation of therapy.

### Which AI Approaches Appear Most Effective by Clinical Task?

The reviewed studies used different methods of AI with different clinical objectives. No single AI method is considered the optimal Ozempic therapy. Instead, effectiveness depends on the clinical task. The supervised ML model that uses EHR data has provided the most evidence regarding patient stratification and predicting treatment response. The DL model that uses images provides the most evidence related to assessing and tracking changes in body composition due to Ozempic therapy. However, they are still mostly in the pilot phase. The adaptive and reinforcement learning models have been shown to have the greatest potential for real-world clinical application when providing for personalized dosing of Ozempic therapy; however, there is currently very limited evidence available for these models, and much of the evidence that exists is not specific to semaglutide. The risk engines and simulation models have also demonstrated the ability to assess long-term cardiovascular and cost-effectiveness of Ozempic therapy, and while they do indirectly assist with making routine dosing decisions, they are generally not sufficient to make these decisions.

### Future Directions

In order to transform AI-driven Ozempic therapy from a proof-of-concept to standard clinical practice, more strategic directions must be followed. First, large-scale multicenter randomized controlled trials (RCTs) involving different patient populations are critical to validating AI-guided dosing algorithms and benchmarking them against standard-of-care regimens. In addition, extended follow-up assessments beyond 24 weeks are necessary to evaluate long-term outcomes such as glycemic durability, cardiovascular protection, and patient adherence. The development of interoperable and standardized data ecosystems, using models such as the Observational Medical Outcomes Partnership common data model [48] and compliance with findable, accessible, interoperable, and reusable (FAIR) principles [49], will be crucial to integrating different data streams, including EHRs, laptops, imaging, and omics. Transparency and explainability in AI models must be prioritized to promote clinician trust, support regulatory paths, and ensure that algorithms can be evaluated rigorously for fairness and reliability. Equally important is the mitigation of algorithmic bias and compliance with regulatory frameworks such as the Food and Drug Administration’s (FDA) Good ML Practice [50], General Data Protection Regulation [51], and the Health Insurance Portability and Accountability Act [52], which ensure fair use across varied T2D populations. Finally, an extensive cost-use analysis is required to determine whether the personalized approach can justify the high cost of semaglutide, thus informing reimbursement strategies and decisions by decision-makers. Collaborating on future innovation with these imperatives will accelerate the integration of AI into Ozempic-based care, improve glycemic control, cardiometabolic health, and general patient quality.

### Ethical and Regulatory Considerations

Despite its promise, AI-enhanced semaglutide therapy must address critical ethical and legal challenges to ensure safe, equitable, and transparent deployment. Key issues include privacy (protecting patient data on a large scale), informed consent (ensuring patients understand how AI informs or overrides physician decisions), algorithmic bias (mitigating underperformance in underrepresented subgroups), and data ownership (clarification of who controls patient information). Explainable AI models are crucial for compliance with bodies such as the FDA [50] and European Medicines Agency [53], reassuring clinicians that dosing recommendations are not “black boxes.” Establishing clear management frameworks and ongoing involvement with patients, clinicians, and decision-makers will maintain public trust as AI-based Ozempic therapy transitions from experimental to mainstream practice.

### Trustworthy AI Considerations

Ensuring that AI models used in Ozempic therapy meet standards of trustworthiness is critical. Future studies must include bias detection protocols, explainability tools (eg, Shapley additive explanations [54] and local interpretable model-agnostic explanations [55]), and rigorous fairness audits to ensure equitable treatment across age, gender, and ethnic groups. Integration with trustworthy data preparation pipelines and alignment with the FDA’s Good Machine Learning Practice for Medical Device Development guidelines [50] and the EU (European Union) Artificial Intelligence Act [56] will be vital for regulatory approval and ethical deployment.

### Limitations

Although many studies emphasize AI’s promise to individualize semaglutide therapy, several restrictions limit current generalizability. Methodological heterogeneity in AI methods (random forests, gradient boosting, convolutional neural networks, k-means, etc) varies widely, complicating direct comparisons and hindering standardized best practices [35,36]. Integration barriers across data sources: merging structured EHR data with unstructured inputs (imaging, wearables, and omics) is still a challenge, and limits extensive predictive modeling [20,31]. Limited long-term evidence: most findings focus on short-term outcomes (12-24 weeks) without exploring extended cardiovascular risk reduction, hospitalization, or mortality [33,36]. Small or homogeneous samples: restrictive sample sizes and limited demographic diversity reduce external validity and increase risk of bias [14,26]. Regulatory and ethical challenges: tools such as d-Nav [32] and logic learning machine [24] face developing supervision, which requires robust explainability and data-privacy compliance. Large-scale RCT deficit: the absence of a sizable, multicenter RCT to confirm the AI-driven semaglutide dose limits direct comparisons with standard regimens. Insufficient long-term follow-up: the persistent effect of AI-defined semaglutide on weight maintenance, cardiovascular risk, and compliance is still unclear due to a lack of multiyear data. Missing cost-use analyses: few studies quantify whether AI-based personalization offsets the high cost of semaglutide, leaving overall economic feasibility unexplored. To address these limitations, especially through large studies and trials, transparent AI models and robust economic evaluations are critical to efficiently integrate AI solutions into routine semaglutide therapy and broader diabetes treatment.

### Conclusion

AI technologies now enable semaglutide therapy to move from fixed, population-level dosing toward data-driven personalization. Evidence from 18 peer-reviewed studies (2019-2025) shows that AI-enhanced regimens deliver greater HbA_1c_ reduction, larger weight loss, and fewer adverse events than standard protocols. High CQAS scores across relevance and clinical-impact domains confirm the maturity of the evidence base, although transparency deficits remain for proprietary or black-box models.

The review introduces 3 advances that have not previously been combined in the semaglutide literature: (1) CQAS benchmarking—a 5-domain rubric that allows objective comparison of methodological quality and clinical use; (2) a translational matrix that links each AI technique to its input data, application domain, and real-world readiness—showing, for example, that reinforcement-learning titration engines are nearest to deployment whereas multiomics integration remains conceptual; and (3) a trustworthy AI-Ozempic framework—a closed-loop architecture that couples predictive analytics, adaptive dosing, and patient-feedback modules to measurable clinical KPIs such as time-in-range, medication persistence, and cardiovascular-event reduction.

Together, these components outline a practical roadmap for precision diabetes care and lay the foundation for scalable digital therapeutics across metabolic disorders.

Validation in multicenter, long-horizon trials; FAIR-compliant, integrative data infrastructures; bias-mitigated and explainable models; and robust cost-use evidence are the critical levers that can convert AI-guided semaglutide from experimental promise into routine care across diabetes and related cardiometabolic diseases.

## Data Availability

The complete, version-controlled codebase used to generate all figures, and supplementary analyses is openly available at GitHub [57].

## Appendix

Multimedia Appendix 1 PRISMA checklist.

## Abbreviations

AI: artificial intelligence
CQAS: Customized Quality Assessment Scale
DL: deep learning
EHR: electronic health record
EU: European Union
FAIR: findable, accessible, interoperable, and reusable
FDA: Food and Drug Administration
GLP-1: glucagon-like peptide-1
HbA1c: hemoglobin A1c
KPI: key performance indicator
MIAGE: Multisource Information Aggregator for Educational and Research Purposes
ML: machine learning
PRISMA: Preferred Reporting Items for Systematic Reviews and Meta-Analyses
RCT: randomized controlled trial
T2D: type 2 diabetes mellitus
